# Further decrease in glycated hemoglobin following ingestion of a LoBAG_30 _diet for 10 weeks compared to 5 weeks in people with untreated type 2 diabetes

**DOI:** 10.1186/1743-7075-7-64

**Published:** 2010-07-29

**Authors:** Mary C Gannon, Heidi Hoover, Frank Q Nuttall

**Affiliations:** 1 Metabolic Research Laboratory and Section of Endocrinology, Metabolism & Nutrition, VA Medical Center, Minneapolis, MN, USA; 2 Department of Food Science & Nutrition, University of Minnesota, St. Paul, MN, USA; 3 Department of Medicine, University of Minnesota, Minneapolis, MN, USA; 4 Nutrition & Food Service, VA Medical Center, Minneapolis, MN, USA

## Abstract

**Background:**

We previously determined that a weight-maintenance, non-ketogenic diet containing 30% carbohydrate (CHO), 30% protein, 40% fat, (30:30:40) (LoBAG_30_) decreased glycated hemoglobin (%tGHb) from 10.8 to 9.1% over a 5 week period in subjects with untreated type 2 diabetes. Both the fasting glucose and postprandial glucose area were decreased. Our objective in the present 10-week study was to determine: 1) whether the above results could be maintained, or even improved (suggesting a metabolic adaptation) and 2) whether the subjects would accept the diet for this longer time period. In addition, protein balance, and a number of other blood and urine constituents were quantified at 5 and at 10 weeks on the LoBAG_30 _diet to address metabolic adaptation.

**Methods:**

Eight men with untreated type 2 diabetes were studied over a 10-week period. Blood was drawn and urine was collected over a 24 hour period at the beginning of the study with subjects ingesting a standard diet of 55% CHO, 15% protein, 30% fat, and at the end of 5 and 10 weeks following ingestion of a LoBAG_30 _diet.

**Results:**

Body weight was stable. Fasting glucose decreased by 19% at week 5 and 28% at week 10; 24-h total glucose area decreased by 27% at week 5 and 35% at week 10 compared to baseline. Insulin did not change. Mean %tGHb decreased by 13% at week 5, 25% at week 10, and was still decreasing linearly, indicating that a metabolic adaptation occurred. Serum NEFA, AAN, uric acid, urea, albumin, prealbumin, TSH, Total T_3_, free T_4_, B_12_, folate, homocysteine, creatinine, growth hormone and renin did not differ between weeks 5 and 10. IGF-1 increased modestly. Urinary glucose decreased; urinary pH and calcium were similar.

**Conclusions:**

A LoBAG_30 _diet resulted in continued improvement in glycemic control. This improvement occurred without significant weight loss, with unchanged insulin and glucagon profiles, and without deterioration in serum lipids, blood pressure or kidney function. Extending the duration of time on a LoBAG_30 _diet from 5 to 10 weeks had little or no further effect on the hormones and metabolites measured, i.e. a metabolic equilibrium was established.

## Background

Our interest over the past several years has been to determine the metabolic consequences in people with type 2 diabetes following ingestion of diets that are lower in carbohydrate and higher in protein than those recommended by government and professional organizations for the general population.

We previously had shown that the increase in blood glucose concentration following a meal is due largely to diet-derived glucose. This includes glucose per se, and that from the hydrolysis of the disaccharides sucrose and lactose. However, it is derived mainly from the hydrolysis of dietary starch. The other monosaccharides important in human nutrition, fructose and galactose, had little effect on the blood glucose concentration [1,2]. We also had shown previously that dietary protein stimulates insulin secretion and either has no effect on blood glucose, or lowers it [3-7].

Since dietary glucose is largely responsible for raising the plasma glucose after a meal, and dietary protein either does not increase the glucose concentration, and indeed may lower it, we have designed diets we refer to as **Lo**w **B**iologically **A**vailable **G**lucose (LoBAG) diets. In these diets we have reduced the carbohydrate content, (particularly, starchy foods) and increased the protein content. The LoBAG diets have been classified according to the per cent of total energy as carbohydrate using a subscript notation. Thus, a 20% carbohydrate diet is referred to as LoBAG_20_, a 30% carbohydrate diet as LoBAG_30_, and a 40% carbohydrate diet is referred to as LoBAG_40_. The protein content in all of the above diets is 30% of food energy, i.e. the protein content is 1 1/2 - 2 fold higher than that in a typical western diet.

All of these diets lowered the integrated 24-hour glucose concentration and resulted in a significant decrease in glycated hemoglobin [8-10].

Our previous studies were designed to be 5 weeks in length because this is the time reported for the glycated hemoglobin (GHb) to decrease by 50% of the ultimate value (t_1/2 _= 33 days) [11] if the plasma glucose concentration is rapidly decreased and is maintained at a lower steady state. The new steady state occurs at ~100 days, the time required for turnover of the red blood cell mass. In the present study we have extended the time of diet intervention from 5 weeks to 10 weeks.

The 24-hour profiles and integrated responses for glucose, insulin, glucagon, non-esterified fatty acid (NEFA), alpha amino nitrogen (AAN), uric acid and urea nitrogen, the creatinine clearance and amount of protein metabolized as well as the a.m. fasting cholesterol, blood pressure, weight, and glycated hemoglobin were determined. In addition, a number of other hormones and metabolites determined in the study are presented.

## Methods

Twelve male subjects were recruited and signed consent forms. They were screened and found to be free of hematologic abnormalities, liver disease, kidney disease, macroalbuminuria (>300 mg/24 h), untreated thyroid disease, congestive heart failure, angina, life-threatening malignancies, proliferative retinopathy, diabetic neuropathy, peripheral vascular disease, serious psychological disorders, and had a body weight <136 kg (300 lb). The inclusion criteria for the study were subjects with type 2 diabetes, c-peptide positive, whose fasting glucose concentration was less than 200 mg/dl (11.1 mmol/L), and who were not taking oral hypoglycemic agents, and had never taken insulin. All subjects had been taking one oral hypoglycemic medication before enrolling in the study. These medications were discontinued for varying periods of time to allow the glycated hemoglobin to stabilize before the study was begun. Diabetes medications were discontinued because the objective of the study was to determine the effect of the LoBAG diet per se, without the confounding effect of a hypoglycemic agent. Allowing the glycated hemoglobin to stabilize before starting the study assured that the maximum effect of the diet would be observed, i.e. the decrease due to the diet was not masked by the increase due to discontinuation of the medication. Other medications being taken by the subjects were continued and remained unchanged during the entire study. The study was approved by the Department of Veterans Affairs Medical Center and the University of Minnesota Committees on Human Subjects.

Eight subjects completed the study. Of the subjects who did not complete the study, 1 subject gave informed consent, but changed his mind before beginning the study; 1 subject chose to discontinue the study after 8 days; 1 subject developed influenza between week 5 and week 10 of the study; 1 subject was discontinued after 5 weeks on the study by the study team and resumed his medication because his %tGHb had not decreased during the preceding 5 weeks. In retrospect, the %tGHb had not stabilized after discontinuing the oral hypoglycemic medication before instituting the diet. It should be added that in none of our previous studies have subjects not completed the study, nor have we discontinued the study for any patient. Patient characteristics are given in Table 1.

**Table 1:** Patient Characteristics

Subject	Age	Ht	Ht	Wt	Wt	BMI	%tGHb	Duration	Concomitant	Medications
		**Inches**	**Cm**	**Lb**	**Kg**			**T2DM**	**Diseases**	

1	67	70	177.8	208.2	94.5	29.9	9.7	3 yr	HT, BPH, osteoarthritis, gout, GERD	terzosin, omeprazole, finisteride, ASA
2	65	70	177.8	165.8	75.3	23.8	11.2	4 yr	HT, TBI	none
3	58	70	177.8	241.2	109.5	34.6	9.3	4 yr	HT, DJD, hyperlipidemia	lisinopril, ranitidine, simvastatin
4	67	65.5	166.4	181.4	82.4	29.8	10.2	4 yr	HT, hyperlipidemia	gemfibrozil, losartan
5	60	71	180.3	185.0	84.0	25.8	9.8	5 yr	HT, hyperlipidemia	calcium/vit D, lisinopril, simvastatin, naproxan, testosterone
6	69	68	172.7	183.4	83.3	27.9	8.6	6 yr	HT, osteoarthritis, TIA, hypercholesterolemia	lisinopril, amlodipine, atenolol
7	48	66	167.6	200.4	92.4	32.8	9.2	2 yr	HT	lisinopril
8	69	70	177.8	206.6	93.8	29.7	11.8	5 yr	HT, BPH, back pain	losartan, naproxan

Abbreviations:

HT: hypertension

BPH: benign prostatic hypertrophy

TBI: traumatic brain injury

DJD: degenerative joint disease

TIA: transient ischemic attack

GERD: gastro-esophageal reflux disease

As indicated previously, a six-day rotating menu was used which was calculated to consist of 30% carbohydrate, 30% protein, 40% fat (10% saturated fat) (LoBAG_30_) [10]. The diet contains foods typical of those consumed by our patient population. Dietary preferences were accommodated whenever possible. All food was provided to the subjects. The total food energy of the diet was individualized to insure that each subject remained weight stable during the study. Our protocol differs from others in the literature using a high protein- low carbohydrate diet ([12], and reviewed in this paper), in that our subjects are weight stable during the study. This was done to eliminate the potential confounding effect of weight loss on blood glucose control.

The protocol used was essentially as described previously [10]. Briefly, blood was drawn for 24-hour profiles of glucose, insulin, C-peptide, glucagon, triacylglycerol, alpha amino acid nitrogen (AAN), non-esterified fatty acids (NEFA), uric acid, urea nitrogen, and creatinine at the beginning of the study with subjects ingesting a control diet of 15% protein, 55% carbohydrate, 30% fat. Thereafter subjects ingested a LoBAG_30 _diet, for which we provided all of the food. At the end of weeks 5 and 10, blood was drawn again for the 24-hour profiles indicated above. During each 24-hour data collection period, urine was collected for determination of microalbumin, calcium, creatinine, glucose, pH, potassium, sodium, urea and uric acid. Glycated hemoglobin was determined weekly. In contrast to the previous 5-week study, the present study was designed to be an intervention only study. A control diet arm was not included in the current study because we had shown previously that a LoBAG_30 _diet improved blood glucose control compared to a control diet [10]

Total glycohemoglobin was measured using boronate-affinity high performance liquid chromatography (Biorad Labs, Hercules, CA). During the study period, the laboratory changed methods to an ion-exchange HbA1c method. A conversion table was developed in order to adjust the ion-exchange data to the boronate affinity method data. Since the majority of the data were obtained with the original method, the data in this paper are reported as total glycohemoglobin. Serum glucose concentrations were determined using a hexokinase method on an Abbott Architect analyzer (Abbott Laboratories, Abbott Park, IL). Serum immunoreactive insulin was determined using an automated chemiluminescent assay on a DPC IMMULITE platform (Diagnostic Products Corp, Los Angeles, CA). Glucagon and C-peptide were determined by radioimmunoassay using kits from Linco Research (a subsidiary of Millipore, Inc. Billerica, MI). The plasma and urine creatinine, plasma urea nitrogen and uric acid were determined with an automated method on an Ortho-Clinical Diagnostics Vitros 950 analyzer (Raritan, NJ). NEFAs were measured enzymically using a kit manufactured by Wako Chemicals (Richmond, VA); total alpha amino nitrogen concentration by the method of Goodwin, which is a measure of the total amino acids; plasma TSH, total triiodothyronine (T_3_) and free thyroxine (T_4_) by Chemiflex (Abbott Architect, Abbott Park, IL), growth hormone (Quest, New Brighton, MN) B_12 _and folate by chemiluminescence (Diagnostic Products, Los Angeles, CA); IGF-1 by RIA (Quest); homocysteine by HPLC (Hewlett-Packard, Palo Alto, CA); microalbumin using a Beckman-Coulter array 360 analyzer (Fullerton, CA); urinary calcium and magnesium colorimetrically on a J & J Vitros Instrument (J & J Engineering, Poulsbo, WA). Weight was determined on a digital scale (Scalitronix, White Plains, NY) in street clothes without shoes. Lean body mass was determined by bioelectric impedance (RJL Systems instrument, Clinton Town, MI). Blood pressure was determined using an automatic Dinemap Instrument (Critikon/Mediq, Pennsauken, NJ).

For quantitation of data obtained over the 24-hour period, all of the net 24-hour incremental area responses were calculated using the overnight fasting value as baseline. Total 24-hour area responses also were calculated, using zero as the baseline. Both area calculations were done using a computer program based on the trapezoid rule [13]. The total amount of protein deaminated was determined by quantifying the urine urea nitrogen excreted over the 24 hours of the study in addition to the amount of urea nitrogen retained endogenously. The latter was calculated by determining the difference in plasma urea nitrogen concentration between the fasting baseline at the beginning and at the end of each 24-hour study period, and correcting for plasma water by dividing by 0.94. In this calculation, it is assumed that there is a relatively rapid and complete equilibration of urea in total body water. Total body water as a percentage of body weight was calculated using the equation of Watson et al. [14]. The overall assumption is that a change in plasma urea concentration is indicative of a corresponding change in total body water urea concentration. In these 24-hour studies, the beginning and ending plasma urea nitrogen concentrations were essentially identical, indicating no retention of urea. The sum of total urea nitrogen in urine and body water was divided by 0.86 to account for 14% being lost to metabolism in the gut [15].

Statistics were determined by Student's t test for paired variates, comparing the results following 10 weeks on the LoBAG_30 _diet to those following 5 weeks on the diet. A P value of <0.01 is the criterion for significance, because of the number of comparisons. Data are presented as means ± SEM. Prism 4 software was used (Graphpad Software, Inc. San Diego, CA) for the iMac computer (Apple, Cupertino, CA).

## Results

### Weight

The subjects' initial mean body weight was 199 lbs (range 165-241 lbs) (90 kg; range 75-109 kg). The mean final weight was 197 lbs (range 166-241 lbs) (89 kg; range 75-109). Thus, the average body weight remained essentially stable during the 10-week study period.

### Glucose

**Figure 1:**
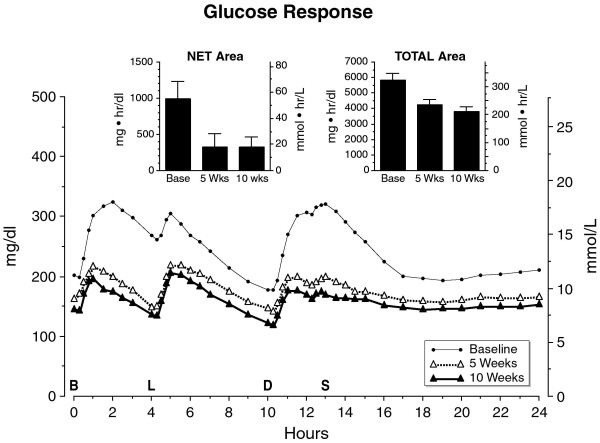
**Mean serum glucose concentration at baseline while on the control diet, (closed circles) and after 5 weeks (open triangles) and after 10 weeks (closed triangles) on the LoBAG_30 _diet**. n = 8 subjects. The letters on the x axis indicate time of ingestion of Breakfast, Lunch, Dinner, and Snack. Inset left - mean net integrated 24-hour area response (using the fasting value as baseline). Inset right - mean total integrated 24-hour area response (using zero as baseline).

The initial mean fasting glucose concentration was 201 ± 13 mg/dl (11.2 ± 0.7 mmol/L) (Figure 1). After five weeks on the diet, the fasting glucose concentration had decreased to 163 ± 12 mg/dl (9.1 ± 0.7 mmol/L). After 10 weeks it had decreased significantly to 145 ± 12 mg/dl (8.1 ± 0.6 mmol/L) (P = 0.02).

**Figure 2:**
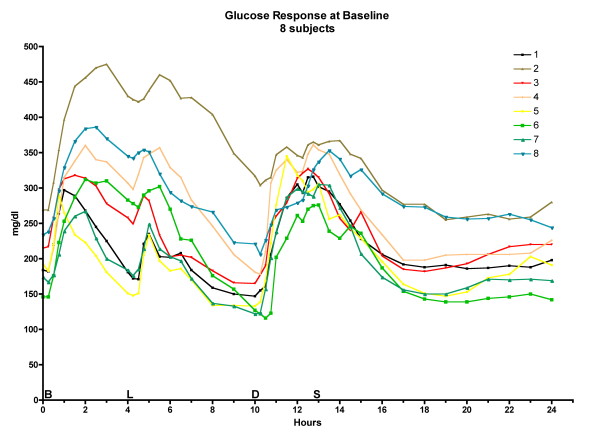
**24-hour glucose response at baseline for 8 individual subjects**. The letters on the x axis indicate time of ingestion of Breakfast, Lunch, Dinner, and Snack.

**Figure 3:**
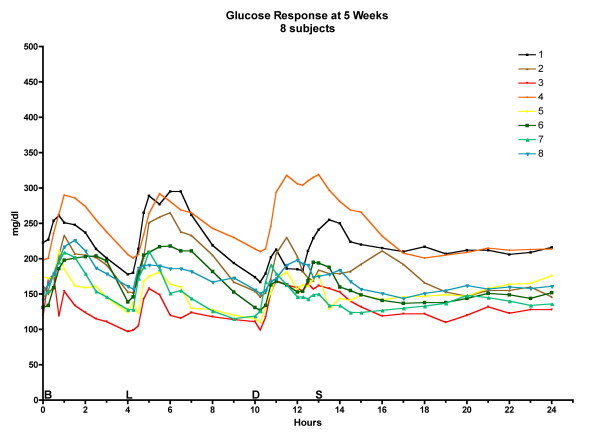
**24-hour glucose response at 5 weeks for 8 individual subjects**. The letters on the x axis indicate time of ingestion of Breakfast, Lunch, Dinner, and Snack.

**Figure 4:**
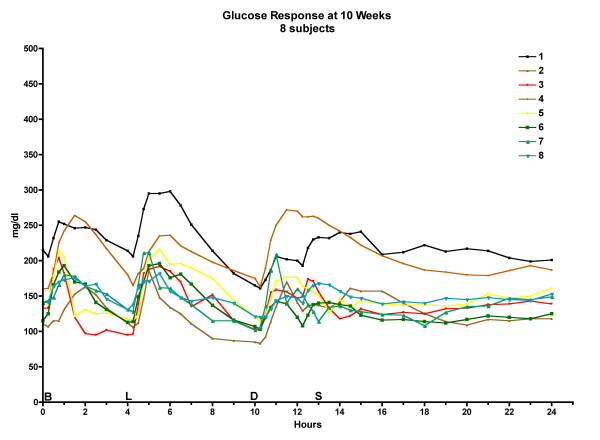
**24-hour glucose response at 10 weeks for 8 individual subjects**. The letters on the x axis indicate time of ingestion of Breakfast, Lunch, Dinner, and Snack.

The postprandial glucose excursions decreased after 5 weeks on the LoBAG_30 _diet, as reported previously [10]. The excursions at 10 weeks were the same as at the 5-week time point. (Please see Figure 2, Figure 3, and Figure 4 for data on individual patients at baseline, week 5, and week 10, respectively.)

The mean net 24-hour integrated glucose area responses, using the fasting value as baseline, were 944 ± 234, 321 ± 185, 326 ± 134 mg•hr/dl (50 ±13; 18 ± 10; 18 ± 7 mmol•hr/L) for the baseline, 5 week and 10 week time periods, respectively. At 5 and 10 weeks this represented a 66 and 65% decrease in net areas, respectively (Figure 1, left inset).

The mean total 24-hour integrated glucose area response was initially 5814 ± 457 mg•h/dl (323 ± 25 mmol•hr/L). After 5 weeks it was 4230 ± 329 mg•h/dl (235 ± 18 mmol•hr/L), a 27% decrease. At 10 weeks it had decreased further to 3805 ± 309 mg•hr/dl (211 ± 17 mmol•hr/L). This represents an additional 10% decrease in response, which was statistically significant (P = 0.03) (Figure 1 insert, right).

### Glycated Hemoglobin

**Figure 5:**
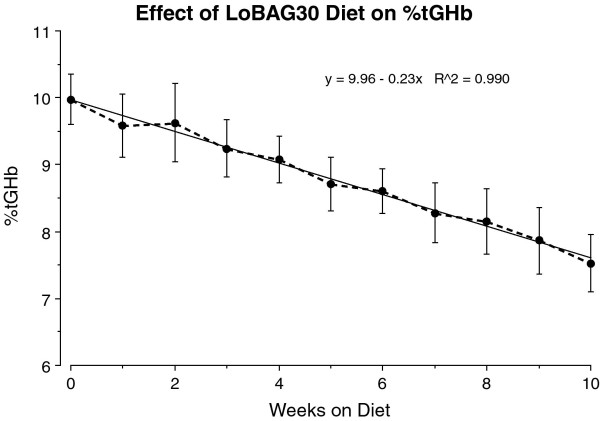
**Mean percentage total glycated hemoglobin (%tGHb) response during the 10 weeks of the LoBAG_30 _diet**.

The mean glycated hemoglobin decreased from 10.0% at baseline to 8.7% at 5 weeks. At the end of 10 weeks it had decreased to 7.5% (P < 0.002) (Figure 5).

### Insulin

**Figure 6:**
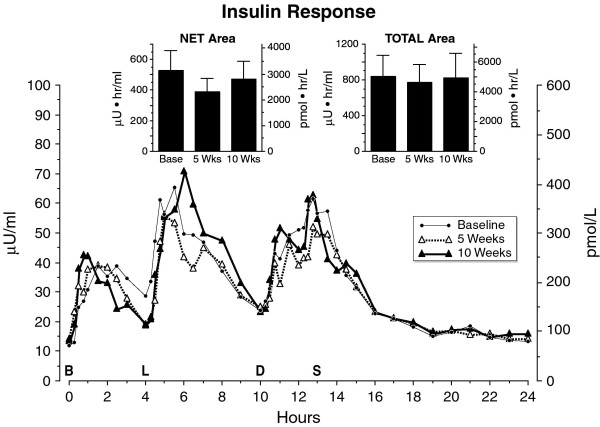
**Mean serum insulin concentration at baseline while on the control diet (closed circles), and after 5 weeks (open triangles) and after 10 weeks (closed triangles) on the LoBAG_30 _diet**. n = 8 subjects. The letters on the x axis indicate time of ingestion of Breakfast, Lunch, Dinner, and Snack. Inset left - mean net integrated 24-hour area response (using the fasting value as baseline). Inset right - mean total integrated 24-hour area response (using zero as baseline).

The mean fasting insulin concentrations were similar before and after 5 weeks and 10 weeks on the diet, as were the excursions after meals (Figure 6). The mean net and total 24-hour integrated insulin areas were essentially the same at baseline, and at 5 and 10 weeks (Figure 6 inserts).

The C-peptide excursions were similar to those of insulin (data not shown).

### Glucagon

**Figure 7:**
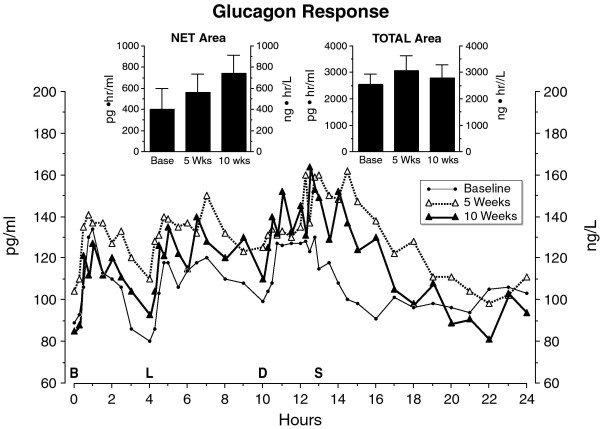
**Mean plasma glucagon concentration at baseline while on the control diet (closed circles), and after 5 weeks (open triangles) and after 10 weeks (closed triangles) on the LoBAG_30 _diet**. n = 8 subjects. The letters on the x axis indicate time of ingestion of Breakfast, Lunch, Dinner, and Snack. Inset left - mean net integrated 24-hour area response (using the fasting value as baseline). Inset right - mean total integrated 24-hour area response (using zero as baseline).

The mean fasting glucagon concentration was not significantly different at baseline, 5 weeks, or 10 weeks after the LoBAG_30 _diet (89 ± 20, 104 ± 19, and 85 ± 18 pg/ml, respectively). The net area response increased progressively from baseline to 5 weeks, and again at 10 weeks, but these increases were not statistically significant. The total glucagon area responses were all similar (Figure 7, insert).

### Triacylglycerol

**Figure 8:**
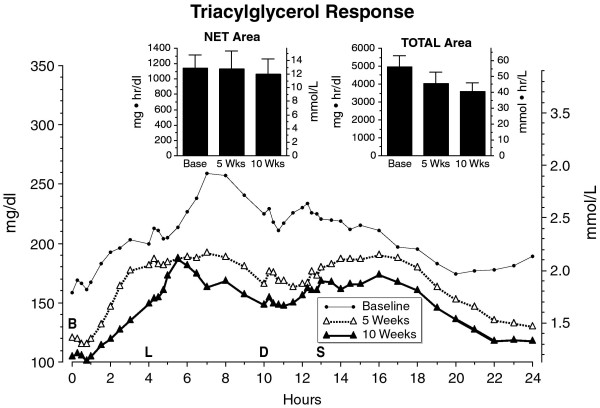
**Mean triacylglycerol concentration at baseline while on the control diet (closed circles), and after 5 weeks (open triangles) and after 10 weeks (closed triangles) on the LoBAG_30 _diet**. n = 8 subjects. The letters on the x axis indicate time of ingestion of Breakfast, Lunch, Dinner, and Snack. Inset left - mean net integrated 24-hour area response (using the fasting value as baseline). Inset right - mean total integrated 24-hour area response (using zero as baseline).

The mean fasting triacylglycerol concentration decreased from a baseline of 159 ± 22 to 119 ± 19 mg/dl at 5 weeks (1.50 ± 0.22 to 119 ± 0.19 mmol/L). It decreased further at 10 weeks to a mean of 105 ± 14 mg/dl (1.05 ± 0.14 mmol/L) (Figure 8). (P = 0.09). The net triacylglycerol area responses were nearly identical. The total triacylglycerol area responses decreased from 4950 ± 640 at baseline to 4039 ± 164 at 5 weeks and 3575 ± 494 mg•hr/dl at 10 weeks (49.5 ± 6.4 at baseline to 40.39 ± 1.64 at 5 weeks and 35.75 ± 494 mmol•hr/L at 10 weeks). The decrease between weeks 5 and 10 approached statistical significance (P = 0.06) (Figure 8, insert).

### NEFA

**Figure 9:**
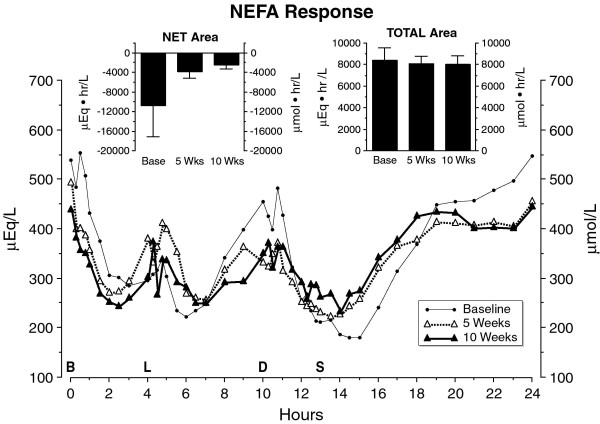
**Mean plasma nonesterified fatty acid (NEFA) response at baseline while on a 55% carbohydrate, 15% protein, 30% fat diet (closed circles), following 5 weeks (open triangles) or following 10 weeks (closed triangles) on a LoBAG_30 _diet**. n = 8 subjects. The letters on the x axis indicate time of ingestion of Breakfast, Lunch, Dinner, and Snack. Inset left - mean net integrated 24-hour area response (using the fasting value as baseline). Inset right - mean total integrated 24-hour area response (using zero as baseline).

The mean fasting NEFA (non-esterified fatty acid) concentration was highest at baseline, lower after 5 weeks and lowest after 10 weeks on the diet (Figure 9). However, these differences were not statistically significant (P = 0.51). A similar decrease in fasting NEFA following ingestion of the LoBAG_30 _diet also was observed in our previous 5-week study [16]. In the 24-hour profile, the NEFA concentrations decreased after meals, as expected, and then transiently increased before the next meal. A nadir was reached after the evening meal, followed by a return to baseline by 0800 the following morning. Both the integrated net area and total 24-hour NEFA area responses were similar following 5 and 10 weeks on the LoBAG_30 _diet (Figure 9, inserts). When the 5- & 10-week net area responses were combined and compared to the baseline area, there were no differences (p value = 0.27).

### NEFA vs Insulin

**Figure 10:**
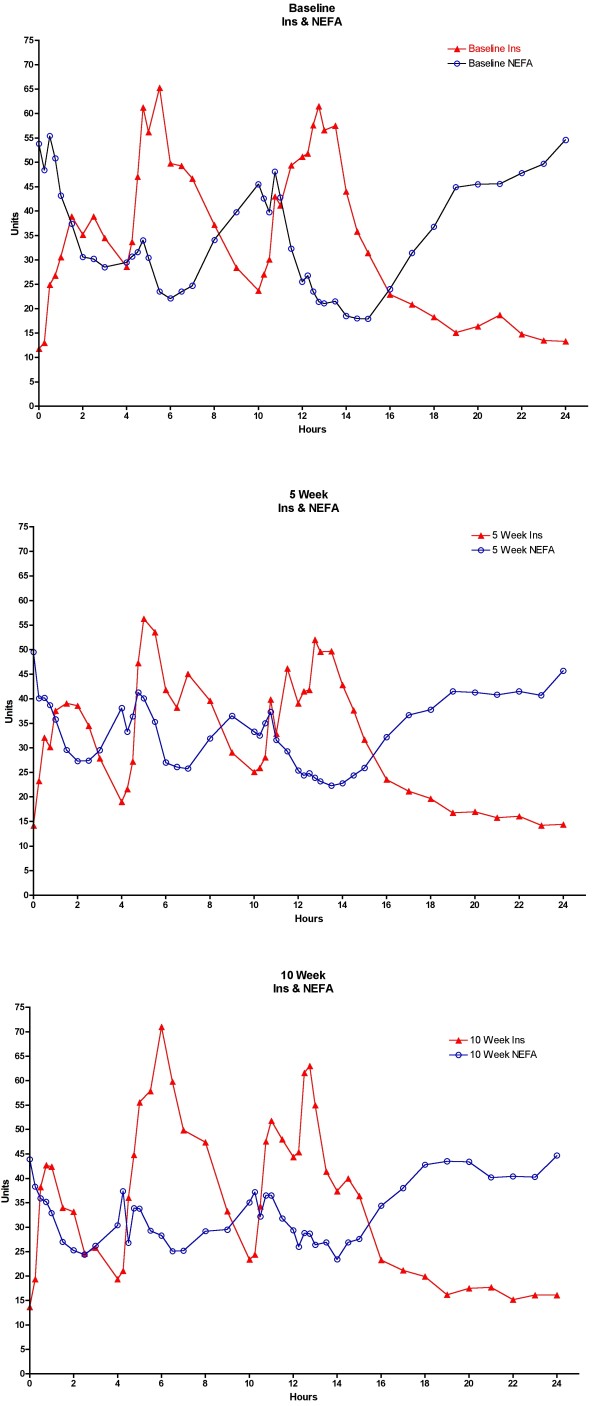
**Relationship between insulin and NEFA concentrations**. Top - Baseline data while on a 55% carbohydrate, 15% protein, 30% fat diet; Middle - following 5 weeks on a LoBAG_30 _diet; Bottom - following 10 weeks on a LoBAG_30 _diet. n = 8 subjects. The letters on the x axis indicate time of ingestion of Breakfast, Lunch, Dinner, and Snack. Units are μU/ml for insulin (closed triangles), and μEq × 10^-1^/L for NEFA (open circles).

The NEFA response following meals is essentially the reciprocal of the insulin response following meals at baseline (Figure 10 top), 5 weeks (Figure 10 middle) and 10 weeks (Figure 10 bottom), as expected.

### AAN

**Figure 11:**
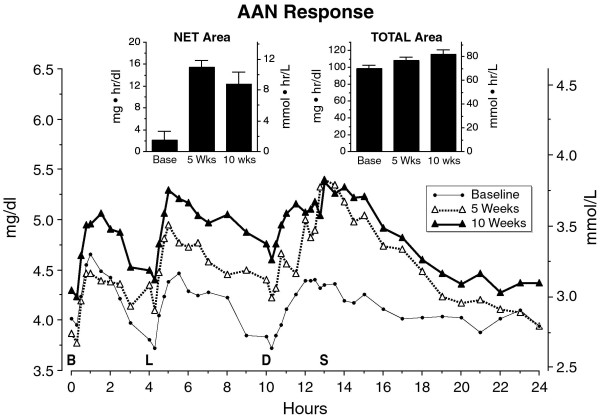
**Mean alpha amino acid nitrogen (AAN, i.e. total amino acid) response at baseline while on a 55% carbohydrate, 15% protein, 30% fat diet (closed circles), following 5 weeks (open triangles) or following 10 weeks (closed triangles) on a LoBAG_30 _diet**. n = 8 subjects. The letters on the x axis indicate time of ingestion of Breakfast, Lunch, Dinner, and Snack.

The mean fasting alpha amino acid nitrogen concentrations were similar before the diet and after 5 weeks or after 10 weeks on the diet (Figure 11). Both the integrated net and total area responses at 5 & 10 weeks were significantly increased compared to baseline (P < 0.01) (Fig. 11, inserts). However, the net and total area responses were similar after 5 and after 10 weeks on the diet.

### Uric Acid

**Figure 12:**
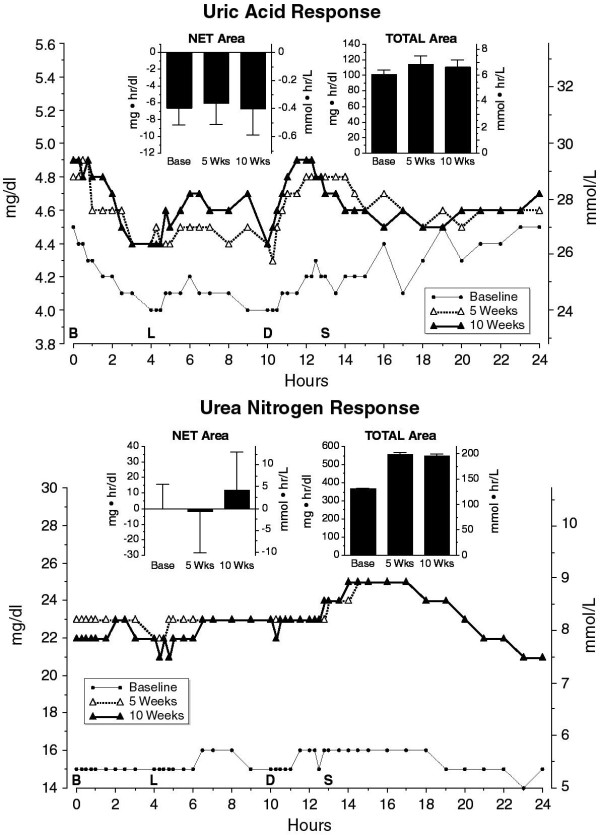
**Top. Mean plasma uric acid response at baseline while on a 55% carbohydrate, 15% protein, 30% fat diet (closed circles), following 5 weeks (open triangles) or following 10 weeks (closed triangles) on a LoBAG_30 _diet**. n = 8 subjects. The letters on the x axis indicate time of ingestion of Breakfast, Lunch, Dinner, and Snack. Inset left - mean net integrated 24-hour area response (using the fasting value as baseline). Inset right - mean total integrated 24-hour area response (using zero as baseline). **Bottom**: Mean urea nitrogen response at baseline while on a 55% carbohydrate, 15% protein, 30% fat diet (closed circles), or following 5 weeks (open triangles) or following 10 weeks (closed triangles) on a LoBAG_30 _diet. n = 8 subjects. The letters on the x axis indicate time of ingestion of Breakfast, Lunch, Dinner, and Snack. Inset left - mean net integrated 24-hour area response (using the fasting value as baseline). Inset right - mean total integrated 24-hour area response (using zero as baseline).

The mean fasting uric acid concentration was modestly, but not significantly higher after 5 or 10 weeks on the diet (p = 0.14) (Figure 12, top). In the 24-hour profiles, the uric acid concentration decreased during the day, regardless of the diet, or the length of time on the LoBAG_30 _diet. The decrease during the day was similar to that noted previously in the 5 week study [16]. The reason for the decrease is unknown, unless it is a dilutional effect. The net 24-hour area was negative, due to the decrease during the day, and was similar at baseline, 5 and 10 weeks (Figure 12, top insert). The total 24-hour areas were not statistically significantly different between baseline, 5 and 10 weeks (Fig. 12 top, insert).

### Urea Nitrogen

The mean fasting plasma urea nitrogen concentration was higher after 5 and 10 weeks on the diet compared to baseline (Figure 12, bottom). The concentrations at 5 and 10 weeks were not different from one another, but were statistically significantly increased compared to baseline (P = 0.004). In the 24-hour profiles, there was a very modest increase in urea nitrogen concentration during the day, with a return to baseline by 0800 the following morning. The responses at 5 and 10 weeks were nearly identical. The net 24-hour urea nitrogen area response was essentially zero at baseline, 5 and 10 weeks (Figure 12, bottom, insert). The 24-hour total area increased by ~50% after 5 and 10 weeks on the LoBAG_30 _diet (Figure 12, bottom, insert). Again, the increases at 5 and 10 weeks were not different from one another, but were statistically significantly increased compared to baseline (p = 0.0001).

### Amount of Protein Ingested and Metabolized

**Figure 13:**
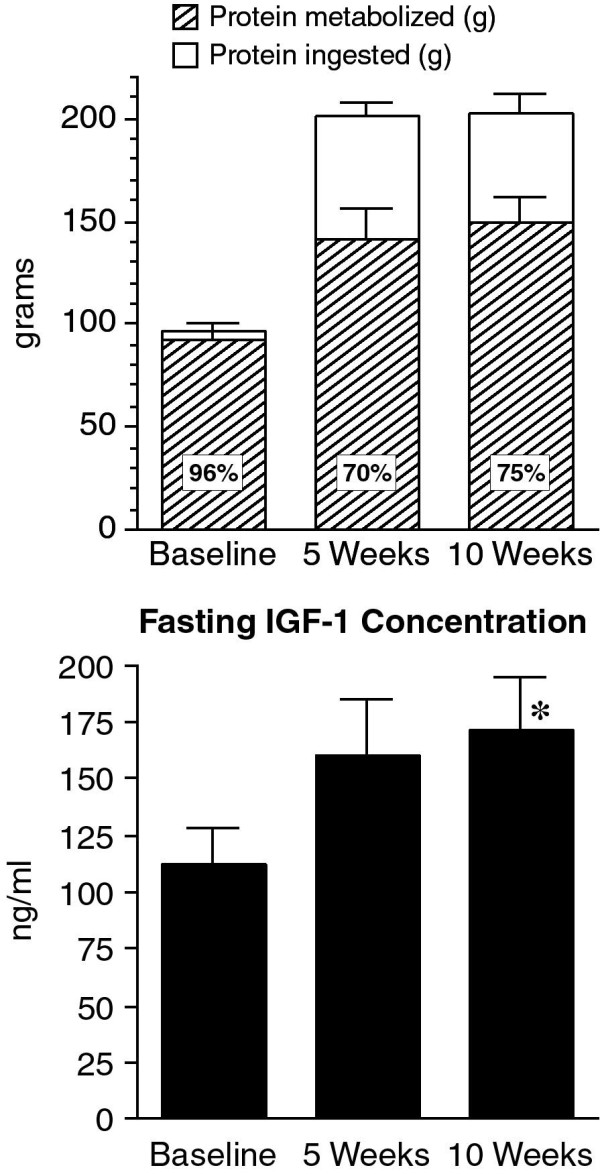
**Top: Protein metabolized and protein ingested**. n = 8 subjects. **Bottom**: Mean fasting (0800 hr) IGF-1 concentration at baseline while on a 55% carbohydrate, 15% protein, 30% fat diet, and 5 weeks and 10 weeks on a LoBAG_30 _diet. n = 8 subjects. * indicates statistical significance P < 0.004.

The calculated total amount of protein ingested during the 24-hour study periods at the beginning and at the end of 5 and 10 weeks was compared with the total protein metabolized (Figure 13, top).

At the beginning of the study, during the first 24 hours while ingesting the control (15% protein) meals, it was calculated that 96 ± 3 g of protein were ingested and 92 ± 5 g were metabolized (96 ± 5%) (Figure 13, top). At the end 5 weeks on the LoBAG_30 _diet, the calculated mean protein ingested over 24 hours was 201 ± 7 g; that metabolized was 141 ± 15 g (70 ± 7%). At the end of 10 weeks, 203 ± 9 g of protein were calculated to have been ingested and 150 ± 12 g were calculated to have been metabolized (75 ± 7%). Thus, the calculated percent of protein metabolized at 5 and 10 weeks after ingestion of the LoBAG_30 _diet was similar and less than that following the control diet.

Calculating the grams of protein metabolized/kg lean body mass, yielded similar results (Baseline = 1.3 ± 0.1, 5 week = 2.0 ± 0.2, 10 week 2.2 ± 0.1 g/kg lean body mass).

### IGF-1

The fasting serum IGF-1 concentration was significantly higher after ingestion of the LoBAG_30 _diet, from a mean at baseline of 112 ± 16 to 160 ± 26 at 5 weeks and to 171 ± 25 ng/ml 10 weeks (Figure 13, bottom). The difference between the 5 and 10-week values was statistically significant (p = 0.01), and both were significantly increased compared to baseline (P ≤ 0.004 and p ≤ 0.0008, respectively).

### Other Hormones and Metabolites

Serum albumin was modestly higher at week 5 compared to baseline or week 10 (Table 2). The increase at week 5 was similar to the statistically significant increase we reported previously [16]. Prealbumin was only modestly higher at week 5 and similar to baseline at week 10. The TSH, total T_3_. Free T_4_, B_12_, folate, homocysteine, growth hormone and renin were similar at baseline, 5 and 10 weeks. None of the minor differences in hormones and metabolites noted above were statistically significant. The fasting total, LDL, HDL cholesterol remained essentially unchanged over the 10 weeks of the study (Table 2). The blood pressure also was unchanged (Table 2).

**Table 2:** Hormones, Metabolites and BP

	Baseline		**5 Week LoBAG**_**30**_		**10 Week LoBAG**_**30**_		P Value
	**Common**	**SI**	**Common**	**SI**	**Common**	**SI**	

Albumin	4.4 ± 0.1 g/dl	44 ± 1 g/L	4.8 + 0.1 g/dl	48 ± 1 g/L	4.5 + 0.1 g/dl	45 ± 1 g/L	0.02
Pre-Albumin	21.3 ± 1.1 mg/dl	21.3 ± 1.1 mg/dl	24.1 + 1.9 mg/dl	24.1 + 1.9 mg/dl	22.3 + 1.5 mg/dl	22.3 + 1.5 mg/dl	0.18
TSH	1.67 ± 0.25 μIU/ml	1.67 + 0.25 mU/L	1.46 + 0.20 μIU/ml	1.46 + 0.20 mU/L	1.59 + 0.24 μIU/ml	1.59 + 0.24 mU/L	0.03
Total T_3_	88.9 ± 4.06 ng/dl	1.37 + 0.06 nM	86.3 + 4.01 ng/dl	1.33 + 0.06 nM	94.0 + 3.51 ng/dl	1.44 + 0.05 nM	0.04
Free T_4_	0.97 ± 0.08 ng/dl	12.5 + 1.03 pM	0.97 + 0.03 ng/dl	12.5 + 0.39 pM	0.93 + 0.05 ng/d	12.0 + 0.64 pM	0.20
B 12	316 ± 45 pg/ml	223 + 33 pM	346 + 49 pg/ml	255 + 36 pM	336 + 53 pg/ml	248 + 39 pM	0.37
Folate	14.9 ± 1.5 ng/ml	33.8 + 3.40 nM	14.9 + 1.5 ng/ml	33.8 + 3.39 nM	13.3 + 1.3 ng/ml	30.1 + 2.95 nM	0.09
Homocysteine	138 ± 13.0 μg/dl	10.2 + 0.96 μM	146 + 10.4 μg/dl	10.8 + 0.77 μM	139 + 18.0 μg/dl	10.3 + 1.33 μM	0.24
Creatinine	1.0 ± 0.03 mg/dl	88.4 + 2.65 μM	1.0 + 0.04 mg/dl	88.4 + 3.54 μM	1.0 + 0.04 mg/dl	88.4 + 3.54 μM	0.09
Growth Hormone	0.2 ± 0.03 ng/ml	0.2 ± 0.03 μg/L	0.2 ± 0.06 ng/ml	0.2 ± 0.06 μg/L	0.3 ± 0.10 ng/ml	0.3 ± 0.10 μg/L	0.16
Renin	0.83 ± 0.34 ng/ml	0.83 ± 0.34 ng/ml	0.99 ± 0.37 ng/ml	0.99 ± 0.37 ng/ml	1.18 ± 0.75 ng/ml	1.18 ± 0.75 ng/ml	0.29
							
**Lipids**							
Total Cholesterol	161 ± 11.8 mg/dl	4.2 ± 0.31 mM	159 ± 16.2 mg/dl	4.1 ± 0.42 mM	156 ± 12.9 mg/dl	4.0 ± 0.33 mM	0.96
LDL-Cholesterol	97 ± 8.5 mg/dl	2.5 ± 0.22 mM	105 ± 14.0 mg/dl	2.7 ± 0.36 mM	103 ± 11.3 mg/dl	2.7 ± 0.29 mM	0.88
HDL-Cholesterol	32 ± 1.7 mg/dl	0.8 ± 0.04 mM	31 ± 0.8 mg/dl	0.8 ± 0.02 mM	32 ± 1.7 mg/dl	0.8 ± 0.04 mM	0.93
							
**BP**							
Blood Pressure (s)		135 ± 4.1 mm Hg		135 ± 5.3 mm Hg		136 ± 4.0 mm Hg	0.99
Blood Pressure (d)		82 ± 2.6 mm Hg		82 ± 3.6 mm Hg		74 ± 2.9 mm Hg	0.15

n = 8

### Urine Data

The total urine volume and sodium excretion were modestly greater at 5 weeks and further increased at 10 weeks compared to baseline (Table 3). Potassium excretion was little changed. Urinary calcium excretion was modestly less after 5 weeks on the diet and remained lower than the baseline value at 10 weeks, but this was not statistically significant.

**Table 3:** 24 Hour Urine

	Baseline		**5 Week LoBAG**_**30**_		**10 Week LoBAG**_**30**_		P Value*
	**Common**	**SI**	**Common**	**SI**	**Common**	**SI**	

Volume	3670 ± 494 ml	3670 ± 494 ml	3811 ± 633 ml	3811 ± 633 ml	4197 ± 689 ml	4197 ± 689 ml	0.11
Sodium	5451 ± 759 mg	237 ± 33 mmol	6417 ± 690 mg	279 ± 30 mmol	7337 ± 1139 mg	319 ± 50 mmol	0.06
Potassium	3822 ± 335 mg	98 ± 8.6 mmol	4017 ± 359 mg	103 ± 9.2 mmol	3900 ± 316 mg	100 ± 8.1 mmol	0.31
Calcium	249 ± 48 mg	6.2 ± 1.2 mmol	210 ± 41 mg	5.3 ± 1.0 mmol	229 ± 33 mg	5.7 ± 0.8 mmol	0.28
Glucose	33.3 ± 13.2 g	185 ± 73 mmol	5.4 ± 3.6 g	30.0 ± 20 mmol	2.8 ± 1.8 g	15.7 ± 9.7 mmol	0.15
Creatinine	1724 ± 113 mg	6.4 ± 1.0 mmol	2109 ± 173 mg	18.7 ± 1.5 mmol	2076 ± 209 mg	18.4 ± 1.8 mmol	0.37
Urea	13.1 ± 0.8 g	0.22 ± 0.01 mol	22.3 ± 1.5 g	0.38 ± 0.03 mol	21.6 ± 1.8 g	0.37 ± 0.03 mol	0.01*
Uric Acid	720 ± 43 mg	4.3 ± 0.3 mmol	971 ± 59 mg	5.8 ± 0.4 mmol	957 ± 75 mg	5.7 ± 0.4 mmol	0.37
pH	6.1 ± 0.1	6.1 ± 0.1	6.2 ± 0.2	6.2 ± 0.2	6.3 ± 0.2	6.3 ± 0.2	0.26
μAlbumin	9 ± 3.4 mg	9 ± 3.4 mg	6 ± 1 mg	6 ± 1 mg	5 ± 0.2 mg	5 ± 0.2 mg	0.13
							
Creatinine Clearance**		117 ± 8.3 ml/min		144 ± 12.1 ml/min		148 ± 11.4 ml/min	0.12

*5 vs 10 weeks

n = 8

** Student's t test on Creatinine Clearance: Baseline vs 5 weeks, p = 0.005; baseline vs 10 weeks, p = 0.002

Urinary glucose excretion decreased significantly after 5 weeks on the LoBAG_30 _diet, as we reported previously, and as expected [16]. It was decreased further after 10 weeks, however the difference between weeks 5 and 10 was not statistically significant.

Urea excretion increased significantly after 5 weeks on the diet, as we reported previously [16]. It decreased very modestly, although significantly at week 10 compared to week 5 (p = 0.01). Uric acid excretion was increased significantly after 5 weeks on the diet, again confirming our previous data [16]. It then decreased modestly, but not significantly at 10 weeks. The urinary pH was essentially constant at all times measured (Table 2).

Only three of the subjects had measurable urinary microalbumin at the beginning of the study. Two of the three subjects urinary micro- albumin was unmeasurable by 10 weeks.

The creatinine clearance did not increase between week 5 and week 10 (Table 3).

## Discussion

As noted previously [10], when subjects consumed the LoBAG_30 _diet for 5 weeks, the fasting glucose concentration was decreased, as was the net integrated glucose area response. In the present study, the subjects ingested foods from the same 6-day rotating menu, but over 10 weeks. The area responses using the fasting value as baseline were the same at 5 and 10 weeks. These similar postprandial responses can be explained by the fact that the meals consumed at 5 and 10 weeks were very similar.

However, the fasting glucose concentration had decreased at 10 weeks when compared to the mean 5 week value. Consequently, the total 24 hr integrated glucose area response was significantly lower at 10 weeks compared to 5 weeks. Thus, a time-dependent metabolic adaptation had occurred in these subjects, but this was exclusively due to a decrease in the overnight fasting glucose concentration. Whether the fasting concentration would continue to decrease if the diet was continued for a longer period of time remains to be determined.

As indicated in the introduction, our previous studies were 5 weeks in duration because this represents the t_1/2 _for the glycated hemoglobin to reflect a decrease in glucose concentration to a new steady state if the change is instantaneous or very rapid [11]. By extending the study to 10 weeks, we were interested in determining whether the %tGHb would indeed continue to decrease as expected. We also were interested in determining if the rate of change between weeks 5 and 10 would be greater than expected based on the reported t_1/2_. If so, this would support the concept that a metabolic change was taking place.

If the change in glycated hemoglobin is an exponential function, the expected decrease at 10 weeks would be 1/2 of that observed at 5 weeks. The %tGHb decreased from 10.0 at baseline to 8.7 at 5 weeks (Δ = 1.3). Thus the predicted value at 10 weeks would be 1/2 of 1.3 or a further decrease of 0.65, i.e. the 10-week value would be 8.05. The actual value was 7.5. Thus, the actual difference at 10 weeks (8.7 - 7.5 = 1.2) is 85% greater than the predicted decrease of 0.65 based on the t_1/2_. Indeed, the rate of change is linear from baseline to week 10 (r^2 ^= 0.99). The reason the decrease in glycated hemoglobin was linear and lower than predicted, we suspect, is because there is a continuing metabolic adaptation in these subjects as a result of a continuing decrease in fasting glucose concentration over the period of the study. This assumes that the change in diet resulted in a very rapid decrease in post-prandial glucoses, but a slower change in the fasting glucose. The latter is likely to be due to a decrease in glucose appearance rate. This is a reasonable expectation, but has not been tested.

Of interest, one of the subjects chose to continue on a similar diet on his own. After 6 months, the %tGHb was continuing to decrease. The initial glycated hemoglobin was 11.2%; at 6 months it was 6.5%, and the final after one year was 6.0%. This occurred without weight loss.

In the present study, the fasting serum insulin concentration changed very little from baseline to week 5, confirming our previous report [10]. The fasting concentration at 10 weeks also was similar. In addition, there was little difference in the postprandial insulin responses at baseline, 5 weeks, and 10 weeks. This is in spite of the fact that the glucose concentrations decreased. Our data suggest that insulin is more efficient in inhibiting glucose production and/or accelerating glucose disposition. The mechanism remains to be determined.

Dietary protein is known to stimulate an increase in circulating glucagon concentration acutely [17]. Of some interest, the glucagon concentration increased progressively throughout the day and did not return to the baseline fasting value until the early morning hours. This pattern is similar to that noted earlier in normal young subjects [18]. However, the return was somewhat delayed when the subjects were consuming the LoBAG_30 _diet. We did observe a progressive increase in postprandial glucagon area responses with the LoBAG_30 _diet [10]. However, the increase did not reach statistical significance in either study. In addition, even though the protein content was increased in the LoBAG_30 _diet compared to the control diet, the total glucagon areas all remained very similar. The metabolic consequences, if any, of the sustained total circulating glucagon concentration is unclear. In any regard, it did not prevent a decrease in circulating glucose. It should be added that we have provided evidence that modest, physiologic elevations in glucagon concentration have little effect on net hepatic glucose output [19].

The fasting triacylglycerol concentration at 5 weeks was 25% less than at baseline. This is modestly less than the 40% decrease we reported previously [10], but the subjects in the current study started with a lower triacylglycerol concentration at baseline (~159 vs. 190 mg/dl). After 10 weeks on the LoBAG_30 _diet, the fasting concentration decreased an additional 12%, again indicating that a metabolic adaptation is occurring in these subjects. Dietary carbohydrate is known to raise the morning fasting triacylglycerol concentration [20]. Our current data, and that reported previously, demonstrate that lowering the carbohydrate content also lowers the 24 hour integrated triacylglycerol concentration.

The NEFA concentration decreased when the insulin concentration increased, as expected. However, a threshold insulin concentration above which the NEFA decreased is not apparent. The general response is that when the insulin concentration increased after meals, the NEFA concentration decreased. As the insulin concentration decreased after the evening meal, the NEFA concentration increased (Figure 7).

In spite of the increased fat content of the LoBAG 30 diet, the lipid profile did not change.

An increase in fasting IGF-1 concentration between baseline and week 5 confirmed our previous data [16]. Indeed, we previously reported that increasing the protein content of the diet from 15% to 30% of total food energy resulted in a 35% increase in IGF-1 concentration, regardless of the carbohydrate:fat ratio of the diet [21,22,16]. In the present study the increase at 5 weeks was 43%. Interestingly, the concentration increased another 7% by week 10, but the rate of increase was slowing, suggesting a near maximal effect of the diet had occurred at this time.

The serum albumin was increased at 5 weeks as we noted previously [16]. However, in the present study, it returned toward baseline at 10 weeks. The difference between the increase at week 5 and the decrease at week 10 approached statistical significance (P = 0.02). There was no reason to suspect a decrease in plasma volume at 5 weeks, or an increase in plasma volume at 10 weeks to explain the changes in albumin concentration. Thus, the return toward baseline could represent another metabolic adaptation to the 30% protein diet.

When the subjects ingested the LoBAG_30 _diet for 5 weeks, the 24-hour urinary urea nitrogen increased, as expected. The mean increase was only ~70% and not 2-fold, as would be expected with a doubling of the protein content of the diet. Interestingly, following an additional 5 weeks on the diet, the urinary urea nitrogen excretion decreased very modestly, but this was statistically significant.

The protein balance, based on the urinary excretion of urea, was positive following 5 weeks on the LoBAG_30 _diet, and was similar to that in our previous study (30% vs 32%). At 10 weeks it was slightly less (25%) (Figure 13). The fate of the nitrogen not excreted in the urine remains unknown, but is consistent. The data indicate that nitrogen was being retained and/or that it was being lost from the gastrointestinal tract in the feces. Loses from any other site would not be sufficient for it to explain the difference. We did not attempt to determine changes in body composition in this study, but an exclusive gain in protein mass also would have to be very large in order to explain the difference.

The creatinine, and uric acid excretion were similar at 5 and 10 weeks, indicating that there was no further adaptation at 10 weeks.

Over the years the concept has developed that a high-protein, "Western" diet would result in a systemic acidosis, result in calcium leaching from bone and increase calcium excretion, all ultimately leading to a decrease in bone mass (Reviewed in [23]). Evidence to refute this concept was published by Bonjour [24]. In the present study the urinary pH was unchanged, as noted by others [24], presumably because of ingestion of fruits and vegetables, which result in an increase in pH. Also, calcium excretion was not increased. Several studies subsequently have reported that an increase in dietary protein has a salutary effect on bone [25-31]. This has been attributed to an increase in IGF-1 (reviewed in [32]), as noted in the present study.

There was no significant difference in the serum NEFA, AAN, uric acid, urea nitrogen, albumin, prealbumin, TSH, Total T_3_, free T_4_, B_12_, folate, homocysteine, creatinine, growth hormone or renin between week 5 and week 10.

The literature on the effects of low carbohydrate diets is increasing rapidly. A Medline search of "low carbohydrate diet" or "Diet, carbohydrate-restricted", limited to humans and English language, yielded 7 papers for the year 2000: 32 for 2005: 81 for 2009. For 2010, through July Week 1, the yield was 26. The majority of this literature contains studies employing weight loss. Although a recent review reported that the beneficial effects of carbohydrate restriction do not require weight loss [33], the references cited [34-36], other than our work, all had different experimental designs or different primary outcome measures than those reported here. Nevertheless, the review points out that data are accumulating to suggest that carbohydrate restriction, without weight loss, potentially has beneficial health effects.

Our studies, including the current 10-week study differ from most in the literature in several respects. First, we make a conscious effort to keep subjects weight stable during the study. Second, we provide all of the food, and we use common foods, not synthetic diets. Also we do not use supplements. Finally, we emphasize keeping a pre-study activity level during the entire period of the study. By including all of these parameters in the experimental design, we have been able to provide sound scientific evidence that not only is blood glucose control improved, evidenced by a decrease in total glycated hemoglobin from 10.0 to 7.5, (Δ2.5%) in the present study, but a metabolic adaptation had occurred during that time that was the result of the dietary intervention.

## Conclusion

In summary, we have extended our study of a LoBAG_30 _diet from 5 weeks to 10 weeks. A further improvement in glycemic control in subjects with untreated type 2 diabetes was observed, indicating a continuing metabolic adaptation. This improvement in glucose control occurred without significant weight loss, with unchanged insulin and glucagon profiles, and without a deterioration in serum lipids, blood pressure or in kidney function. A LoBAG_30 _diet ingested for 10 weeks resulted in a modest further increase in IGF-1. Urinary glucose excretion decreased further at 10 weeks, as would be expected with a continuing decrease in fasting glucose and %tGHb. Twenty-four hour profiles of serum NEFA, AAN, uric acid and urea nitrogen were unchanged at 10 weeks compared with 5 weeks. The per cent dietary protein accountable as urinary nitrogen daily was only 68% at 5 weeks and only ~75% at 10 weeks. The fate of the remaining ingested protein remains to be determined. A number of other blood and urine constituents of interest were unchanged. Lastly, the subjects were able to ingest a 30% carbohydrate, 30% protein, 40% fat diet for 10 weeks without difficulty. Indeed, some subjects preferred it.

This provides further evidence to support the concept that a LoBAG diet should be beneficial for some subjects with type 2 diabetes.

## Competing interests

The authors declare that they have no competing interests.

## Authors' contributions

MCG and FQN contributed equally to the study i.e. experimental design, obtaining funding, analyzing data and writing the manuscript. HH planned the menus and met with the patients twice each week during the study.

All authors have read and approved the final manuscript.
